# Development of TACE Refractoriness Scores in Hepatocellular Carcinoma

**DOI:** 10.3389/fmolb.2021.615133

**Published:** 2021-03-26

**Authors:** Li Chen, Chen-Xi Yu, Bin-Yan Zhong, Hai-Dong Zhu, Zhi-Cheng Jin, Guang-Yu Zhu, Qi Zhang, Cai-Fang Ni, Gao-Jun Teng

**Affiliations:** ^1^ Center of Interventional Radiology and Vascular Surgery, Department of Radiology, Zhongda Hospital, Medical School, Southeast University, Nanjing, China; ^2^ Department of Interventional Radiology, the First Affiliated Hospital of Soochow University, Suzhou, China

**Keywords:** hepatocellular carcinoma, transarterial embolization refractory, risk factors, nomogram, overall survival

## Abstract

**Purpose:** To identify the independent risk factors for transarterial embolization (TACE) refractoriness and to develop a novel TACE refractoriness score and nomogram for predicting TACE refractoriness in patients with hepatocellular carcinoma (HCC).

**Methods:** Between March 2006 and March 2016, HCC patients who underwent TACE monotherapy as initial treatment at two hospitals formed the study cohort and validation cohort. The criteria of TACE refractoriness followed the Japan Society of Hepatology 2014 version of TACE refractoriness. In the study cohort, the independent risk factors for TACE refractoriness were identified, and TACE refractoriness score and nomogram were then developed. The accuracy of the systems was validated externally in the validation cohort.

**Results:** In total, 113 patients from hospital A formed the study cohort and 122 patients from hospital B formed the validation cohort. In the study cohort, 82.3% of the patients (*n* = 93) developed TACE refractoriness with a median overall survival (OS) of 540 days (95% CI, 400.8–679.1), and the remaining 20 patients in the TACE-non-refractory group had a median OS of 1,257 days (95% CI, 338.8–2,175.2) (*p* = 0.019). The median time for developing TACE refractoriness was 207 days (95% CI, 134.8–279.2), and a median number of two TACE procedures were performed after refractoriness developed. The independent risk factors for TACE refractoriness were the number of tumors and bilobular invasion of HCC. TACE refractoriness scores <3.5 indicated a lower incidence of TACE refractoriness, whereas scores >3.5 points indicated a higher incidence (*p* < 0.001). In the validation cohort, 77.9% of the patients (*n* = 95) developed TACE refractoriness with a median OS of 568 days (95% CI, 416.3–719.7), and a median OS of 1,324 days was observed in the TACE-non-refractory group (*n* = 27; 95% CI, 183.5–2,464.5).

**Conclusions:** TACE refractoriness impairs the OS of HCC patients. The number of tumors and bilobular invasion status were independent risk factors for TACE refractoriness. The TACE refractoriness score can be an effective tool and easy approach to predict the risk of TACE refractoriness status.

## Introduction

Hepatocellular carcinoma is a major health problem worldwide and is especially more commonly seen in the developing countries or regions (Forner et al., 2018). Nearly 80% of HCC in China is first diagnosed at the mid-late stage due to the asymptomatic features of early HCC (Zhou et al., 2018). According to the Barcelona Clinic Liver Cancer (BCLC) staging system (Llovet et al., 1999; Forner et al., 2010), transarterial chemoembolization is recommended as the first-line treatment for patients at intermediate stage (BCLC B) (1). The global HCC BRIDGE (Bridge to Better Outcomes in HCC) study, a multiregional large-scale longitudinal cohort study including 18,031 patients from 14 countries, proved TACE the most widely used approach for HCC across BCLC stages from intermediate to advanced stages (Park et al., 2015).

In general, overall survival in HCC patients treated with TACE reaches 70.3% at 1 year, 51.8% at 2 years, 40.4% at 3 years, and 32.4% at 5 years (Lencioni et al., 2016). Patient’s general condition, underlying liver function, tumor response, tumor stage, and treatment technique are factors that can largely influence the treatment outcomes (Bruix, 2002; Xu et al., 2015; Kim et al., 2016).

For the reasons above, the concept of TACE refractoriness has recently drawn much attention. Japan Society of Hepatology defined TACE refractoriness/TACE failure in 2010 and updated in 2014 (Kudo et al., 2011; Kudo et al., 2014) as 1) intrahepatic lesion, which consisted of 1) two or more consecutive insufficient responses of the treated tumor (viable lesion >50%) even after changing the chemotherapeutic agents and/or reanalysis of the feeding artery seen on response-evaluation computed tomography (CT)/magnetic resonance imaging (MRI) at 1–3 months after having adequately performed selective TACE; 2) two or more consecutive progressions in the liver (the number of tumors increased compared to that before the previous TACE procedure) even after having changed the chemotherapeutic agents and/or reanalysis of the feeding artery seen on response-evaluation CT/MRI at 1–3 months after having adequately performed selective TACE; 2) continuous elevation of tumor markers immediately after TACE even though a slight transient decrease is observed; 3) appearance of vascular invasion; or 4) appearance of extrahepatic spread. The International Expert Panel on Interventions in Hepatocellular Carcinoma (EPOIHCC) defines TACE refractoriness as no response after ≥3 TACE procedures within a 6 month period to the same area (Cheng et al., 2014). Since then, several studies found that TACE refractoriness had a significant impact on prognosis (Kudo, 2011; Sieghart et al., 2013; Song et al., 2013; Hiraoka et al., 2015). Therefore, identifying the risk factors for TACE refractoriness and developing a model for predicting prognosis of patients with TACE refractoriness are crucial for HCC patients before being treated with TACE. The purpose of this study was to identify the independent risk factors for TACE refractoriness and to develop a TACE refractoriness score and nomogram for predicting TACE refractoriness in HCC patients receiving TACE monotherapy as initial treatment.

## Patients and Methods

### Patients

**FIGURE 1:**
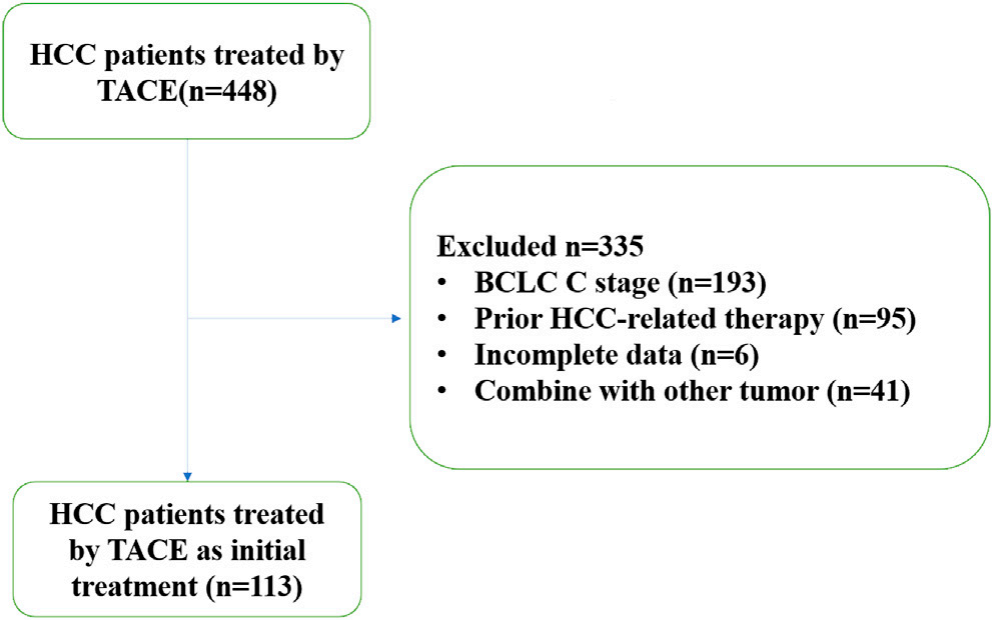
Patient selection. HCC: hepatocellular carcinoma; TACE: transarterial chemoembolization.

Between March 2006 and March 2016, patients with HCC undergoing TACE as an initial treatment at Hospital A were retrospectively studied to form the study cohort (Figure 1). From April 2007 to December 2016, patients with HCC who received TACE as initial treatment at Hospital B were retrospectively reviewed as the external validation cohort. The diagnosis of HCC was made as per the diagnosis criteria of European Association for the Study of the Liver (EASL, 2018) Clinical Practice Guidelines: Management of hepatocellular carcinoma (2018 Edition) (2018): 1) in patients with cirrhosis, multiphase enhanced CT or MRI shows hallmark signs of HCC with an α-fetoprotein (AFP) concentration ≥400 μg/L; and 2) in noncirrhotic patients, diagnosis of HCC should be confirmed by pathology.

### Inclusion and Exclusion Criteria

The inclusion criteria were as follows: 1) age between 18 and 90 years; 2) liver function was suitable for TACE treatment (Child-Pugh A or B level); 3) general condition was suitable for TACE treatment [Eastern Cooperative Oncology Group (ECOG) performance scores = 0]; 4) BCLC stage A or B; and 5) no previous HCC-related treatment. The exclusion criteria were: 1) severe coagulation dysfunction that could not be corrected; 2) severe liver dysfunction (Child-Pugh C) or irreversible liver decompensation; 3) extrahepatic metastasis or vascular invasion; 4) ECOG scores >2; 5) insufficient follow-up data; and 6) history of any other tumor.

### Study Objectives

The primary outcome measure was the occurrence of TACE refractoriness, which was defined according to the criteria of the JSH and the Liver Cancer Study Group of Japan (LCSGJ) (Kudo et al., 2014). The second outcome measure was OS. Baseline characteristics, including tumor size, number of tumors, diameter of the largest lesion, extent of tumor, unilobular or bilobular invasion, and vascular invasion, were collected by two experienced radiologists from multiphase enhanced CT or MRI. Assessment of tumor response after TACE was completed based on the modified Response Evaluation Criteria in Solid Tumors (Lencioni and Llovet, 2010). Clinical data, such as performance status, hepatitis history, cirrhosis history, and ascites, were collected. Serology of AFP, bilirubin, albumin, and white blood cell count were collected as laboratory data.

### Treatment Procedure

TACE was performed within 2–3 days after diagnosis of HCC as described. Using the Seldinger technique, an arterial catheter (5-Fr) was inserted into the femoral artery after local anesthesia. The catheter was then advanced in the hepatic artery, and digital subtraction angiography was performed. The tumor-feeding vessels were superselected using the catheter or microcatheter (2.8-Fr) to infuse a suspension containing 20–60 mg of doxorubicin hydrochloride (Adriamycin; Shenzhen Main Luck Pharmaceutical Inc., Shenzhen, China) and 2–20 ml of iodized oil (Lipiodol Ultra-Fluide; Laboratoire Guerbet, Roissy-Charlesde Gaulle, France). Gelfoam sponge embolization was performed following iodized oil embolization. The dosages of doxorubicin and iodized oil were determined by the patient’s liver function and tumor characteristics.

### Follow-Up

According to the TACE on-demand schedule (Terzi et al., 2012; Zhong et al., 2017), the patients were followed up by dynamic enhanced CT or MRI 4–6 weeks after the procedure. If the lesion had a good response, then no additional treatment was given, and patients were asked to undergo follow-up CT/MRI and α-fetoprotein evaluation in 2–3 months; otherwise, a repeated TACE session was considered. Subsequent TACE was not necessary if patients reached either of the following endpoints: 1) complete devascularization of the lesion; or 2) liver function not suitable for additional TACE procedure. In these situations, patients were recommended to receive other treatments, such as sorafenib, iodine-125 seed implantation, and supportive therapy. At each follow-up, the patients’ imaging study, laboratory data, and general condition were carefully reviewed.

### Statistical Analysis

All data were analyzed using SPSS 21.0 for Windows (IMB Corporation, Somers, NY, United States). The categorical variables, such as sex, unilobular or bilobular invasion, BCLC staging, and vascular invasion status, were compared using the χ^2^ test or Fisher’s exact test. Continuous variables with a normal distribution, such as the number of tumors, tumor diameter, and serum AFP, were compared using the *t* test. A logistic regression model and a neural network were used to identify the independent prognostic factors for TACE refractoriness. A nomogram was estimated based on the conclusion of logistic regression model and by the package of rms in R version 3.0.2. A *p*-value of <0.05 was considered statistically significant.

### Establishment of TACE Refractoriness Score and Nomogram

The TACE refractoriness scoring system and nomogram were established based on the identified significant risk factors by univariate analyses. The accuracy of the nomogram was evaluated by the concordance c statistic (C index) in the validation cohort. The C index was assessed using the receiver operating characteristic curve analysis. The C index of the prognostic models is usually between 0.6 and 0.85 (Royston et al., 2009).

## Results

### Clinical Characteristics of Patients

A total of 113 patients at Hospital A and 122 at Hospital B were enrolled in the study cohort and validation cohort, respectively. The baseline characteristics of the patients in both cohorts were shown in Table 1-3. No significant difference was observed in baseline characteristics between the TACE-refractory and TACE-non-refractory groups in either the study cohort or validation cohort. Some differences in the baseline characteristics were detected between these two hospitals, including BCLC stage, sex, hepatitis infection status, and liver function. The validation cohort was used to evaluate the accuracy of TACE refractoriness score, so it does not interfere with further analysis.

**TABLE 1:** Baseline characteristics in the study cohort.

Characteristics	TACE-refractory group (*n* = 93)	TACE-non-refractory group (*n* = 20)	*p* Value
Age, yr, mean (range)	61.33 (33–89)	66.30 (36–90)	0.129
Sex (male/female)	83/10 (89%)	18/2 (90%)	1.000
Hepatitis B (yes/no)	67/26 (72%)	17/3 (85%)	0.273
PS (0/1)	65/28 (70%)	16/4 (80%)	0.425
TBIL (μmol/L), mean ± SD	18.19 ± 12.97	16.93 ± 8.64	0.680
ALB (g/L), mean ± SD	36.28 ± 5.31	37.74 ± 5.02	0.263
AST (IU/L), mean ± SD	51.05 ± 62.10	48.25 ± 34.43	0.846
ALT (IU/L), mean ± SD	40.01 ± 36.46	45.10 ± 25.25	0.554
WBC (10^9^/L), mean ± SD	5.75 ± 2.23	6.09 ± 2.87	0.547
Hb (g/L), mean ± SD	134.56 ± 18.77	135.05 ± 18.62	0.916
BCLC stage (A/B)	20/73 (22%)	9/11 (45%)	0.029
HKLC stage (I/II/III)	21/32/40	7/9/4	0.133
TN, mean ± SD	3.34 ± 1.61	2.05 ± 1.23	0.001
TD, cm, mean ± SD	7.26 ± 4.31	7.02 ± 3.99	0.819
BI (yes/no)	47/46 (51%)	1/19 (5%)	<0.001

PS: physical status; TBIL: total bilirubin; ALB: albumin; AST: aspartate transaminase; ALT: alanine aminotransferase; WBC: white blood cell count; TN: tumor number; TD: tumor diameter; BI: bilobular invasion; SD: standard deviation.

**TABLE 2:** Baseline characteristics in the validation cohort.

Characteristics	TACE-refractory group (*n* = 95)	TACE-non-refractory group (*n* = 27)	*p* Value
Age, yr, mean (range)	59.42 (27–92)	60.41 (36–85)	0.716
Sex (male/female)	77/18 (81%)	19/8 (70%)	0.287
Hepatitis B (yes/no)	53/42 (56%)	18/9 (66%)	0.380
PS (0/1)	75/20 (79%)	18/9 (66%)	0.206
TBIL (μmol/L), mean ± SD	17.37 ± 8.51	20.58 ± 9.07	0.081
ALB (g/L), mean ± SD	39.44 ± 5.02	37.91 ± 4.36	0.167
AST (IU/L), mean ± SD	79.56 ± 205.70	65.33 ± 61.34	0.482
ALT (IU/L), mean ± SD	63.61 ± 153.07	50.32 ± 40.18	0.788
WBC (10^9^/L), mean ± SD	6.24 ± 2.55	5.42 ± 1.88	0.123
Hb (g/L), mean ± SD	131.95 ± 20.64	133.44 ± 19.14	0.736
BCLC stage (A/B)	1/94 (1%)	3/24 (11%)	0.034
HKLC stage (I/II/III)	11/40/44	7/13/7	0.074
TN, mean ± SD	3.44 ± 1.09	2.89 ± 1.16	0.032
TD, cm, mean ± SD	8.20 ± 4.20	6.45 ± 3.50	0.051
BI (yes/no)	34/61 (36%)	6/21 (22%)	0.247

PS: physical status; TBIL: total bilirubin; ALB: albumin; AST: aspartate transaminase; ALT: alanine aminotransferase; WBC: white blood cell count; TN: tumor number; TD: tumor diameter; BI: bilobular invasion; SD: standard deviation.

**TABLE 3:** Baseline characteristics between the study and validation cohorts.

Characteristics	Study cohort (*n* = 113)	Validation cohort (*n* = 122)	*p* Value
Age, yr, mean (range)	62.21 (33–90)	59.64 (27–92)	0.125
Sex (male/female)	101/12 (89%)	96/26 (79%)	0.033
Hepatitis B (yes/no)	84/29 (74%)	71/51 (58%)	0.013
PS (0/1)	81/32 (72%)	93/29 (76%)	0.459
TBIL (μmol/L), median	17.97 ± 12.3	18.08 ± 8.7	0.409
ALB (g/L), mean ± SD	36.54 ± 5.27	39.1 ± 5.05	<0.001
AST (IU/L), mean ± SD	50.56 ± 58.05	76.41 ± 183.62	0.004
ALT (IU/L), mean ± SD	40.91 ± 34.70	60.67 ± 136.3	0.022
WBC (10^9^/L), mean ± SD	5.52 ± 2.26	6.06 ± 2.43	0.434
Hb (g/L), mean ± SD	134.65 ± 18.66	132.28 ± 20.25	0.354
BCLC stage (A/B)	29/84 (26%)	4/118 (3%)	<0.001
HKLC stage (I/II/III)	28/41/44	18/53/51	0.144
TN, mean ± SD	3.12 ± 2.62	3.32 ± 1.12	0.423
TD, cm, mean ± SD	7.21 ± 4.24	7.81 ± 4.11	0.279
BI (yes/no)	48/65 (36%)	40/82 (22%)	0.139

PS: physical status; TBIL: total bilirubin; ALB: albumin; AST: aspartate transaminase; ALT: alanine aminotransferase; WBC: white blood cell count; TN: tumor number; TD: tumor diameter; BI: bilobular invasion; SD: standard deviation.

### Kaplan-Meier Survival Curves of Overall Survival

**FIGURE 2:**
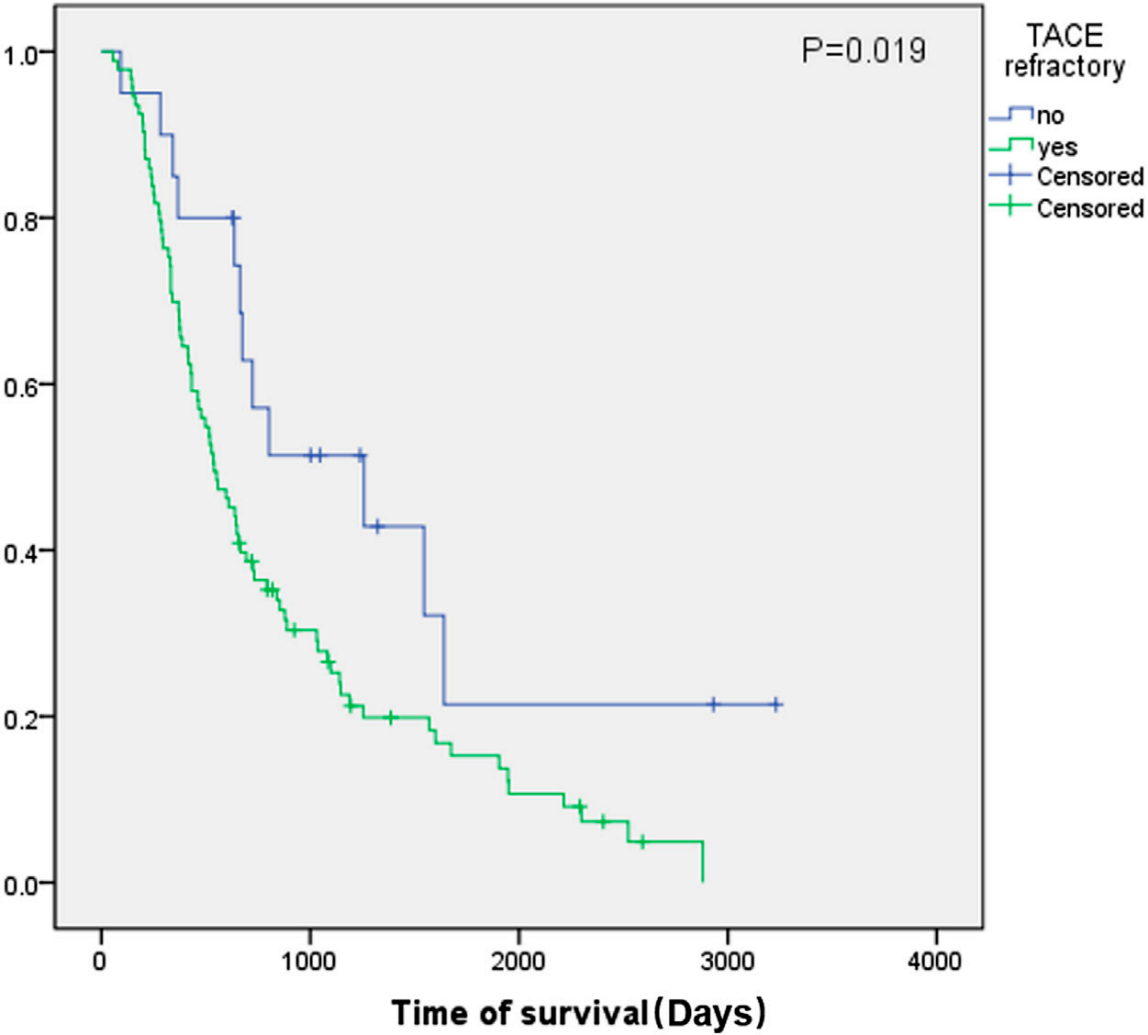
Kaplan-Meier survival curves of the study cohort. Green line: TACE-refractory group. Blue line: TACE-non-refractory group.

Kaplan-Meier curves were generated for different groups in both the study and validation cohorts. In the study cohort, 82.3% (*n* = 93) of the patients were TACE-refractory with a median OS of 540 days (95% CI, 400.8–679.1). In the TACE-non-refractory group (*n* = 20), the median OS was 1,257 days (95% CI, 338.8–2,175.2). The log-rank test showed a significant difference between these two groups (*p* = 0.019) (Figure 2).

**FIGURE 3:**
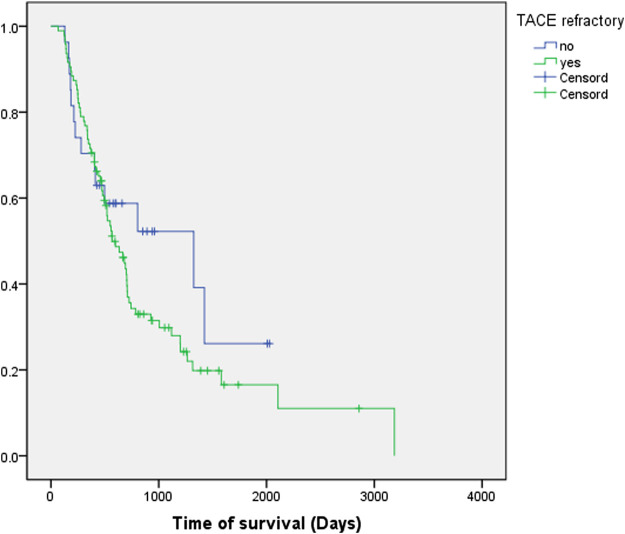
Kaplan-Meier survival curves of the validation cohort. Green line: TACE-refractory group. Blue line: TACE-non-refractory group.

In the validation cohort, 77.9% (*n* = 95) of the patients were TACE-refractory with a median OS of 568 days (95% CI, 416.3–719.7), while the median OS was 1,324 days (95% CI, 183.5–2,464.5) in the TACE-non-refractory group (*n* = 27) (*p* = 0.300) (Figure 3).

### Univariate and Neural Network Analysis

**FIGURE 4:**
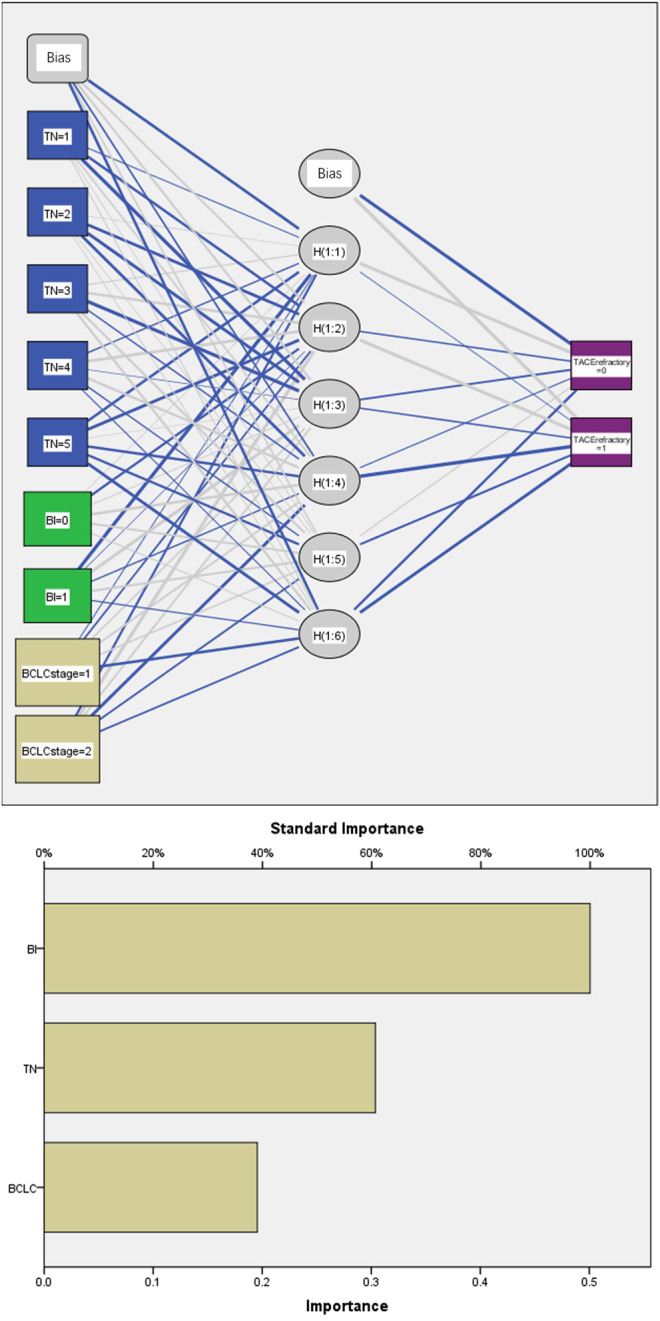
Neural network analysis. BI: bilobular invasion; TN: tumor number. There were six nodes in the hidden layers. A total of 70% of samples were selected for training and the remaining 30% for validation.

The results of univariate analysis showed that the number of tumors (*p* = 0.001) and bilobular invasion (*p* < 0.001) had significant effects on TACE refractoriness. Multi-Layer Perceptron was also used to establish a neural network analysis, with the input variates being the number of tumors, bilobular invasion, BCLC stage, history of hepatitis, and serology indices and output variate being TACE refractoriness. A total of 70% of samples were selected for training, and the remaining 30% were assigned for validation. The outcome of neural network showed the number of tumors and bilobular invasion as top two important indices for TACE refractoriness (Figure 4).

### Logistic Regression Analysis and Development of TACE Refractoriness Score

The number of tumors and bilobular invasion were entered into a logistic regression analysis (Table 4). Both indices remained significant independent prognostic factors for TACE refractoriness. The calculated regression coefficients (B-values) were tripled and rounded to facilitate the calculation of the TACE refractoriness score (Table 4). The TACE refractoriness score was the sum of points given to these two variables. This scoring system identified two subgroups with distinct prognoses. Patients with a TACE refractoriness score of 0–3.5 points had a low incidence of TACE refractoriness, whereas those with a score >3.5 points faced a high incidence of TACE refractoriness (*p* < 0.001). The prognostic performance of TACE refractoriness score was verified in the validation cohort by concordance c statistic. The C-index of this model was 0.80 (95% CI, 0.70–0.90) in the study cohort and 0.65 (95% CI, 0.52–0.78) in the validation cohort.

**TABLE 4:** Multivariate analysis of prognostic factors of the study cohort.

Variable		Multivariate analysis			TACE refractoriness Score
		OR	95% CI	*p* Value	
TN		1.465	1.183–1.815	0.001	TN×1
BI	No	1.0			0
	Yes	12.572	1.556–101.565	0.018	7.5

TN: tumor number, BI: bilobular invasion.

### Prognostic Nomogram for TACE Refractoriness

**FIGURE 5:**
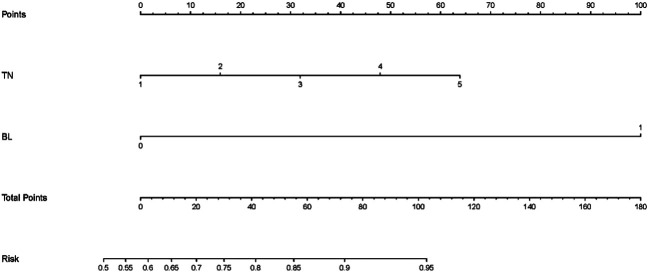
Nomogram for TACE refractoriness. TN: tumor number; BI: bilobular invasion. According to a patient’s condition, locate the patient’s point on the TN/BI axis and draw a line upwards to find the corresponding point. The sum of these two points (variables) can then be located on the total points axis, and a line is to be drawn downwards to match the likelihood of developing TACE refractoriness.

The prognostic nomogram was created to estimate the possibility of TACE refractoriness based on both significant independent factors (Figure 5). In this nomogram, each variable axis displayed a point of an individual patient. The sum of points reflected the likelihood of developing TACE refractoriness.

## Discussion

TACE has been the first-line treatment for HCC patients at the intermediate stage. TACE refractoriness, as a signal of poor response, has gained tremendous interest in recent years (Kim et al., 2012; Sieghart et al., 2013; Johnson et al., 2016). It was first described by the JSH in 2010 and then updated in 2014. Later, the EPOIHCC and EASL published their own definitions of TACE refractoriness in 2011 (Park et al., 2013) and 2014 (Raoul et al., 2014), respectively. In this study, the JSH 2014 definition of TACE refractoriness was chosen because it has been widely accepted by radiologists for relatively easy application. As the earliest definition released, many researchers referred to this version for TACE refractoriness, and examples include Hiraoka A. et al. (Hiraoka et al., 2015) and Lee S. et al. (Lee et al., 2017). In addition, the JSH 2014 definition of TACE refractoriness was practical in clinical settings. Radiologists could easily interpret the TACE refractoriness status based on CT and serum AFP results after patients received two consecutive, adequate TACE procedures.

Our study demonstrated that the OS of the TACE-refractory group was shorter than that of the TACE-non-refractory group in both the study cohort (540 vs. 1,257 days, *p* = 0.019) and validation cohort (568 vs. 1,324 days). These data indicated that TACE refractoriness, which was associated with the number of tumors and bilobular invasion, could contribute to poor prognosis and impair patients’ OS (Johnson et al., 2016; Lee et al., 2017).

Similar to many studies, the number of tumors contributed to TACE refractoriness as an independent risk factor in the present work. Many staging systems that use the number of tumors as a crucial index include the Cancer of The Liver Italian Program (CLIP) scoring system, the Chinese University Prognostic Index (CUPI) system, the International Cooperative Study Group on Hepatocellular Carcinoma (ICSGOHC) simple staging system, and the BCLC staging system (Llovet et al., 1999; Llovet and Bruix, 2000; Leung et al., 2002; Vauthey et al., 2002). In the CLIP scoring system, uninodular lesion is worth 0 point with median survival time being 36 months, while multinodular is worth one point with median survival time of 22 months. The CUPI system and the ICSGOHC simplified staging system were based on the TNM staging system, where a single tumor was classified as T1, T2, and T3a and multiple tumors classified as T3b, T3c, and T4. In the BCLC staging system, a single lesion or three lesions with diameter smaller than 3 cm are considered stage A and recommended to be treated by curative methods.

In the current study, bilobular invasion was strongly associated with TACE refractoriness. This finding was also supported by the studies of Lladó, L et al. (Lladó et al., 2000) and Hiraoka, A et al. (Hiraoka et al., 2006) in which bilobular invasion was proved to have prognostic value. The former study included 143 patients treated with TACE and used univariate analysis to clarify the significant effect of bilobular invasion (*p* = 0.04). The latter study published in 2006 depicted the association between bilobular invasion and poor prognosis (*p* < 0.05) in 121 patients.

This present study reported and validated a TACE refractoriness scoring system and a nomogram to predict the occurrence of TACE refractoriness. The tools can be useful in predicting the likelihood of TACE refractoriness by the number of tumors and presence of bilobular invasion. In this scoring system, each tumor counts one point, and bilobular invasion counts 7.5 points. Patients with a TACE refractoriness score over 3.5 points suffered a high incidence of TACE refractoriness. Therefore, this score can act as a simple tool to predict whether the patient suffers a high risk of TACE refractoriness. In the nomogram, the number of tumors and presence of bilobular invasion are assigned with individual points, and their sum becomes the total score to predict the likelihood of TACE refractoriness.

For those who are prone to be TACE-refractory, molecular-targeted therapies such as sorafenib and lenvatinib are recommended by various guidelines (Kudo et al., 2011; Park et al., 2013; Kudo et al., 2014). Some retrospective studies showed the efficacy of sorafenib to prolong OS and time to progression (TTP) (Ikeda et al., 2014; Ogasawara et al., 2014). Ogasawara, S. et al. compared the use of sorafenib and TACE in their study and discovered that both OS (25.4 vs. 11.5 months) and TTP (22.3 vs. 7.7 months) were significantly longer in the sorafenib group than in the TACE group. In the study by Arizumi, T., a similar conclusion was made as OS in the sorafenib and TACE groups was 24.7 and 13.6, respectively (Arizumi et al., 2015).

The main limitation of this study is the relatively small sample size. For this reason, only two variables were included in the TACE refractoriness scoring system and nomogram. The second limitation is that the retrospective nature of this study may have caused recalling bias during the follow-up. Lastly, the baseline characteristics between the two hospitals were not balanced, which could make our conclusion more difficult to understand. However, it proved, on the other hand, that our TACE refractoriness scoring system and nomogram can provide high accuracy for patients with different baseline data.

## Conclusion

In conclusion, this study showed that TACE refractoriness may impair OS of patients with HCC. The number of tumors and presence of bilobular invasion were independent risk factors for TACE refractoriness. The successfully developed TACE refractoriness scoring system and nomogram can serve as simple but effective tools for predicting the occurrence of TACE refractoriness before the first TACE procedure. Patients with a TACE refractoriness score >3.5 points are at a higher risk of TACE refractoriness.

## Data Availability

The original contributions presented in the study are included in the article/Supplementary Material, further inquiries can be directed to the corresponding author.
